# Novel Mutations in Putative Nicotinic Acid Phosphoribosyltransferases of *Mycobacterium tuberculosis* and Their Effect on Protein Thermodynamic Properties

**DOI:** 10.3390/polym14081623

**Published:** 2022-04-18

**Authors:** Yu-Juan Zhang, Muhammad Tahir Khan, Madeeha Shahzad Lodhi, Hadba Al-Amrah, Salma Saleh Alrdahe, Hanan Ali Alatawi, Doaa Bahaa Eldin Darwish

**Affiliations:** 1 Chongqing Key Laboratory of Vector Insects, Institute of Entomology and Molecular Biology, College of Life Sciences, Chongqing Normal University, Shapingba, Chongqing 401331, China; 2 Institute of Molecular Biology and Biotechnology (IMBB), The University of Lahore, 1 KM Defence Road, Lahore 58810, Pakistan; madeeha.shahzad@imbb.uol.edu.pk; 3 Department of Biological Sciences, Faculty of Science, King Abdulaziz University, Jeddah 221589, Saudi Arabia; hggaber@kau.edu.sa; 4 Department of Biology, Faculty of Science, University of Tabuk, Tabuk 71491, Saudi Arabia; salrdahe@ut.edu.sa (S.S.A.); ddarwish@ut.edu.sa (D.B.E.D.); 5 Department of Biological Sciences, University College of Haqel, University of Tabuk, Tabuk 71491, Saudi Arabia; halatwi@ut.edu.sa; 6 Botany Department, Faculty of Science, Mansoura University, Mansoura 35511, Egypt

**Keywords:** *pncB1*, mutations, TB, drug resistant, *Mycobacterium tuberculosis*

## Abstract

*pncB1* and *pncB2* are two putative nicotinic acid phosphoribosyltransferases, playing a role in cofactor salvage and drug resistance in *Mycobacterium tuberculosis*. Mutations have been reported in first- and second-line drug targets, causing resistance. However, *pncB1* and *pncB2* mutational data are not available, and neither of their mutation effects have been investigated in protein structures. The current study has been designed to investigate mutations and also their effects on *pncB1* and *pncB2* structures. A total of 287 whole-genome sequenced data of drug-resistant *Mycobacterium tuberculosis* isolates from Khyber Pakhtunkhwa of Pakistan were retrieved (BioSample PRJEB32684, ERR2510337-ERR2510445, ERR2510546-ERR2510645) from NCBI. The genomic data were analyzed for *pncB1* and *pncB2* mutations using PhyResSE. All the samples harbored numerous synonymous and non-synonymous mutations in *pncB1* and *pncB2* except one. Mutations Pro447Ser, Arg286Arg, Gly127Ser, and delTCAGGCCG1499213>1499220 in *pncB1* are novel and have not been reported in literature and TB databases. The most common non-synonymous mutations exhibited stabilizing effects on the *pncB1* structure. Moreover, 36 out of 287 samples harbored two non-synonymous and 34 synonymous mutations in *pncB2* among which the most common was Phe204Phe (TTT/TTC), present in 8 samples, which may have an important effect on the usage of specific codons that may increase the gene expression level or protein folding effect. Mutations Ser120Leu and Pro447Ser, which are present in the loop region, exhibited a gain in flexibility in the surrounding residues while Gly429Ala and Gly127Ser also demonstrated stabilizing effects on the protein structure. Inhibitors designed based on the most common *pncB1* and *pncB2* mutants may be a more useful strategy in high-burden countries. More studies are needed to elucidate the effect of synonymous mutations on organism phenotype.

## 1. Introduction

Over recent years, the misuse and irregular intaking of antibiotics in tuberculosis (TB) treatment caused the emergence of resistant MTB [1,2,3,4]. Although several of studies explored potent anti-tuberculosis targets [5,6], the molecular modifications in the core structure of proteins have significantly enhanced its antimicrobial characteristics [7].

Although genes encoding NAD salvage-specific enzymes are present, de novo synthesis of MTB NAD can only show up-regulation of the salvage pathway genes under hypoxia. In cofactor salvage, the two putative nicotinic acid phosphoribosyltransferases, *pncB1* and *pncB2*, play an important role. NAD starvation of the de novo pathway mutant shows a bactericidal effect. Inhibitors against NAD synthetase common to recycling pathways and also de novo synthesis exhibited the same bactericidal effect against nonreplicating and actively growing MTB isolates. These investigations highlighted the role of *pncB1* and PncB2 in the universal pathway and as potential targets for active latent TB [8].

Preiss-Handler is a recycling pathway present in a variety of microbes containing nicotinate phosphoribosyltransferases (*pncB*) and the universal pathway enzymes, *nadD* (nicotinic acid mononucleotide adenylyltransferase) and *nadE* (NAD synthetase) [9]. *pncB1* appears to play a role in basal NAD levels, whereas *pncB2* is regulated in vivo growth and hypoxia [8]. The occurrence of two genes of *pncB* in the MTB genome is an interesting mystery as *M. leprae* lacks *pncB* genes and has lost these components of the recycling pathway during extensive deletion. *M. smegmatis* encodes only one *pncB* in the genome.

*pncB2* plays an important role in the adaptation to nonreplicating persistence including hypoxia [10], nitric oxide [11], and starvation [12]. The up-regulation of the *pncB2* gene expression throughout hypoxia and enhanced salvage pathway activity confirms the important role of NAD salvage in human granulomas. In fact, *pncB2* also reportedly remains a member of the DosR regulon [11]. In our previous study [13], we reported mutations in *pncB1* and *pncB2*; however, the studied samples were very few and the details of the relationships in the mutations were not studied.

Here, in the current investigation, whole-genome sequences of drug-resistant MTB isolates were analyzed to observe the frequency of mutations in *pncB1* and *pncB2* genes. The mutations’ frequency and their possible effects on protein structure from experimentally determined whole-genome sequences submitted to the NCBI were also determined in all amino acid positions of *pncB1* and *pncB2* proteins.

## 2. Materials and Methods

### 2.1. Whole-Genome Sequence Data Retrieval

Seventy-eight drug-resistant WGS from the same geography in FASTQ format were downloaded from the NCBI genome (ERX3360434-ERX3360514, ERR2510337-ERR2510445, ERR2510546-ERR2510645) to analyze the genomic variation in *pncB1* and *pncB2*. According to the laboratory information, random samples were collected from 25 districts of KPK. All the samples were subjected to genomic reprocessing.

### 2.2. Whole-Genome Sequence Analysis

The quality of the sequence was checked with FASTQC to trim the low-quality raw reads and the genome was mapped against the reference strain H37rv (NC_000962.3) [14] using PhyResSE, a reliable and simple server for *Mycobacterium tuberculosis* WGS analysis. Genomic data of Illumina Next-Generation Sequencing and Ion Torrent were analyzed in paired or single-end reads. PhyResSE applied methods from FastQC, BWA, SAMtools, and QualiMap. In-depth QC was performed and applied mapping performance and reports of antibiotic resistance, lineage, and mutations were generated in VCF and CSV file format [15].

### 2.3. Structure Modeling of pncB1

The crystal structure of nicotinate phosphoribosyltransferases (*pncB1*) is not available in Protein Data Bank (PDB), a database containing the three-dimensional structures of proteins and nucleic acids [16]. Therefore, the 3D structure was retrieved from the AlphaFold structure database (P9WJI9) [17].

### 2.4. Ramachandran Plot of pncB1 Modelled Structure

To validate the protein structures, the Ramachandran plot is one of the most important tools, showing the φ/ψ angles mapping pairs of the polypeptide backbone in the form of “allowed” or expected values. The modeled structure was subjected to PROCHECK to validate that the amino acids residues have modelled correctly. Ramachandran plot outliers have been considered as the standard of protein structure analysis [18,19]. The modeled structure of *pncB1* was also validated using a protein structure analysis (ProSA) server [20] to predict the z score available online.

### 2.5. Mutations Effect on pncB1 Stability

To check whether the non-synonymous mutations have any effect on *pncB1* protein stability and flexibility, a point mutation was created in the specific position in the DynaMut server and the mutant was subject to the DynaMut server [21]. The server is very useful to infer the substitution effects on protein structure stability, using vibrational entropy changes in wild type and mutants. The server uses graph-based signatures to measure the impact of a mutation on the structure. This approach is performing well with accurate prediction (*p*-value < 0.001).

### 2.6. Secondary Structure Prediction of Wild Type and Mutant

Secondary structures of wild type and mutants have been predicted using the PSIPRED server that allows the users to submit the sequence. The server is highly accurate for protein secondary structure prediction [22]. To evaluate the performance, PSIPRED used a stringent validation approach achieving an average Q3 score of 76.5% which is the highest-level accuracy for any methods published to date.

## 3. Results

Among the 287 WGS, 230 harbored mutations in *pncB1*, 36 contain mutations in *pncB2* and 21 samples remained wild type for both of these gene mutations (Supplementary Files S1 and S2, Supplementary Materials). These samples harbor mutations either in *pncB1* or *pncB2* (Supplementary Files S1), among which the most common detected in *pncB1* are Pro447Ser (ccg/Tcg), Gly429Ala (ggc/gCc) and eight nucleotide deletions at genomic position 1499213–1499220 (Table 1). Mutations Pro447Ser, Arg286Arg, Gly127Ser, and delTCAGGCCG 1499213>1499220 in *pncB1* are novel and have not been reported in literature and TB databases including GMTV. Very few samples harbor mutations in other locations of the protein, i.e., Ser120Leu (tca/tTa), Arg286Arg (cgg/cgC), and Gly127Ser (ggc/Agc).

In total, 36 out of 287 samples harbored 34 synonymous and two non-synonymous mutations in *pncB2* (Table 2). The most common mutation found was Phe204Phe (ttt/ttC), present in eight genomic samples. Although all these mutations were synonymous, such genomic isolates still need further validation for phenotypic effect. Mutations Ala323Val (gcg/gTg) and Phe286Val (ttc/Gtc) were also novel.

**Figure 1:**
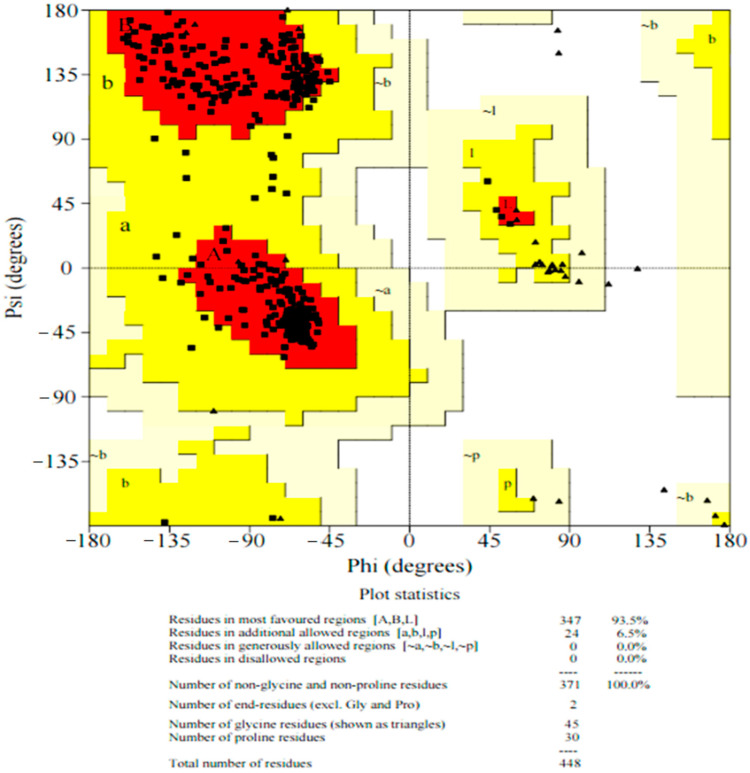
Ramachandran plot of modeled structure nicotinate phosphoribosyltransferases. Among 371 amino acids, 347 (93.5%) residues have been modeled in the favorable regions and 6.5% in the allowed region.

Ramachandran plot of modeled *pncB1* seems highly accurate with 93% (347 amino acids) in the favorable region and 6.5% (24 residues) in the allowed region (Figure 1). The mutation effect was predicted on the modeled structure of *pncB1* through the DynaMut server.

**Figure 2:**
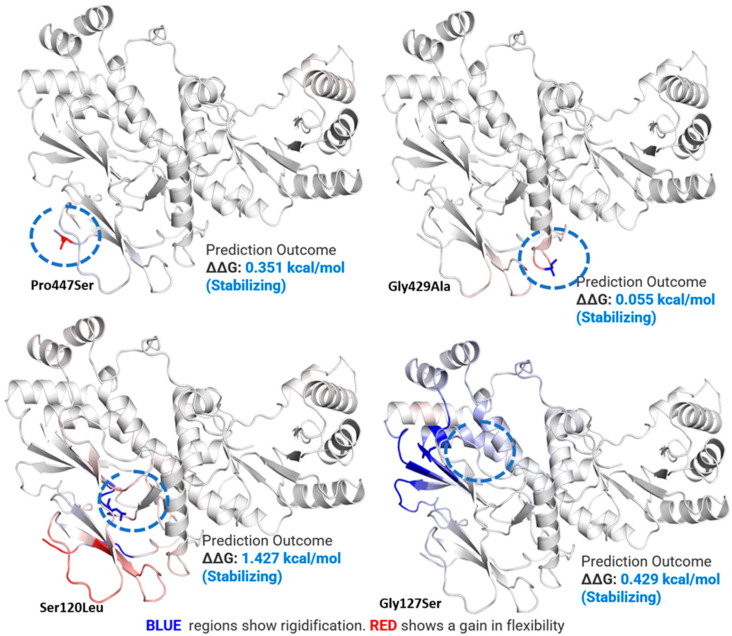
Mutation effect on the *pncB1* structure. The location of the mutations is encircled in blue. These mutations are present in the loop regions. Mutations Ser120Leu and Pro447Ser are present in the loop region and exhibited a gain in flexibility in the surrounding residues (red). Gly429Ala and Gly127Ser also demonstrated stabilizing effects on the protein structure.

The most common non-synonymous mutations in *pncB1* demonstrated a stabilizing effect (Figure 2) which may be useful for MTB survival and growth in extreme conditions. According to flexibility analysis, the mutant Ser120Leu seems a little more flexible in some amino acids but not in all locations. The remaining mutant showed very little rigidification of amino acid residues in *pncB1*.

Both Ser120Leu and Pro447Ser mutations, which have been detected in the loop region of *pncB1*, exhibited a gain in flexibility and stabilizing effect (Δ0.351 kcal/mol). Mutation Pro447Ser, in which a hydrophobic residue is mutated into polar amino acid, exhibiting a stabilizing effect, is present in the loop region. This change exhibited a limited effect on the surrounding amino acid residues.

**Figure 3:**
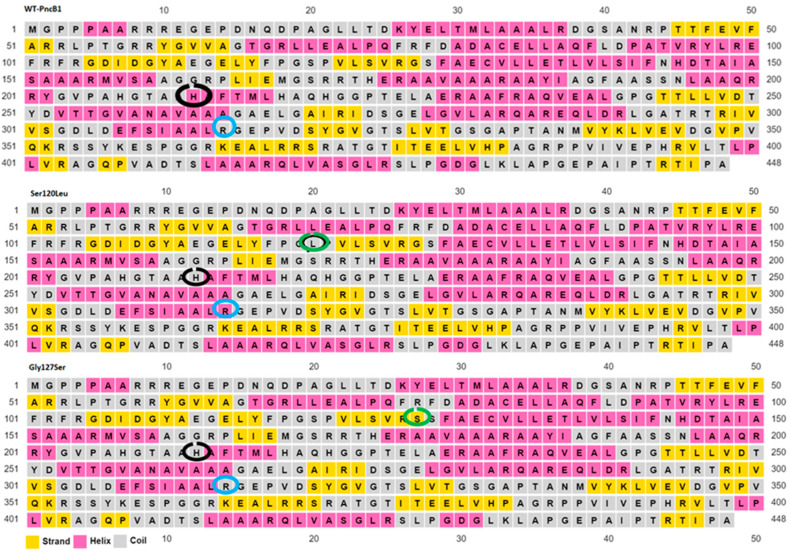
Secondary structure of wild type (WT) and mutant *pncB1* protein. Secondary structure was predicted using the PSIPRED server. The position of the mutations is shown with a green circle. Residues with black circles changed into loop mutants when compared with WT.

**Figure 4:**
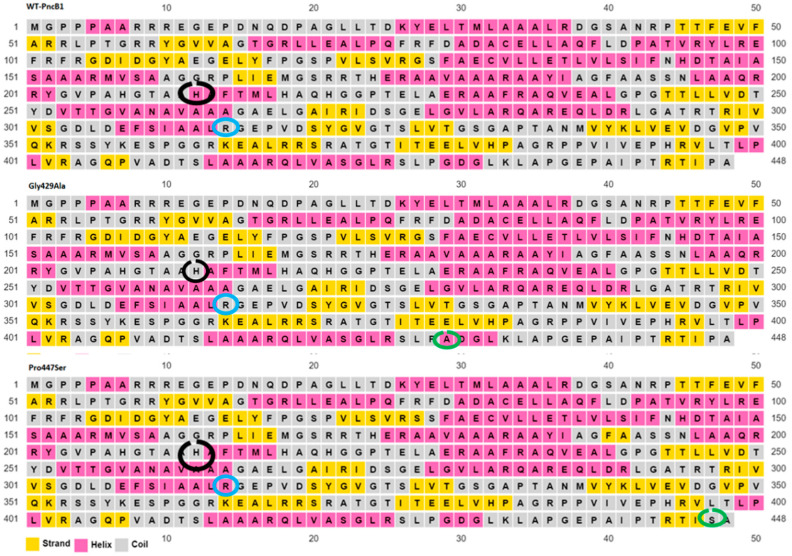
WT and mutant *pncB1* protein secondary structure. This structure has Helices 18 and 23 strands. The position of the mutations is shown with a green circle while a black circle shows the histidine residues that changed into a loop in mutant *pncB1*.

**Figure 5:**
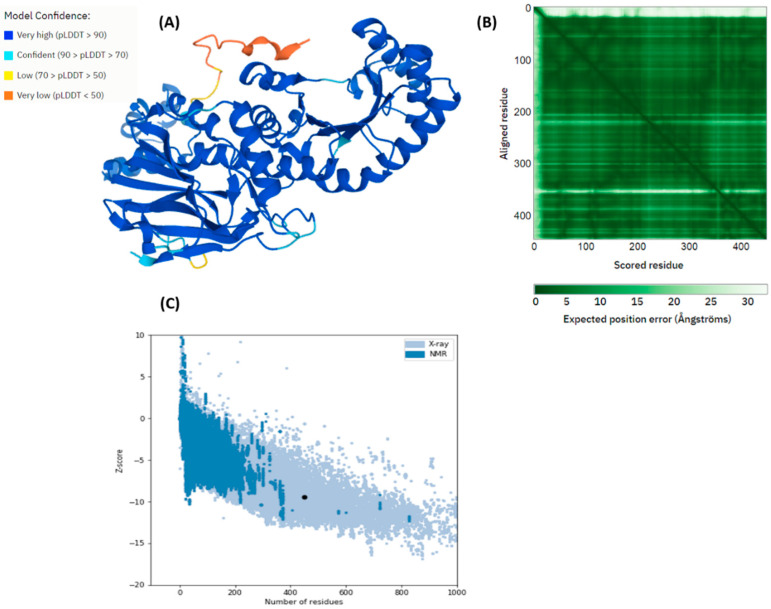
*pncB1* model validation. (**A**) AlphaFold per-residue confidence score (pLDDT). (**B**) The color at position (x, y) indicates AlphaFold’s expected position error at residue x, when the predicted and true structures are aligned on residue y. (**C**) ProsA validation modeled *pncB1* (Z-score −9.44).

Mutations at position S120L and G127S in *pncB1* caused the histidine residues at position 211 to be a part of the loop region when compared with the wild type (Figure 3). Similarly, arginine at position 305 which is a part of the loop (circled blue) in WT, G127S, and G429A (Figure 4), changed into a helix in mutants S120L and P447S. Structure validations of *pncB1* has been shown in Figure 5, showing a Z score −9.44. 

## 4. Discussion

The roles of *pncB1* and *pncB2* are obvious in the NAD synthetase pathway and the antimicrobial activity of potential inhibitors against *pncB1* and *pncB2* of MTB isolates may have a therapeutic effect on the treatment of non-replicating isolates in latent TB. However, designing inhibitors based on *pncB1* and *pncB2,* the most common mutants circulating in the field, may be a more useful strategy in high-burden countries. In our previous study [13], we reported mutations in *pncB1* and *pncB2*; however, comprehensive details of the relationships in the mutations were not studied. In a recent study, *pncB1* was linked with pyrazinamide (PZA) resistance through the analysis of lineage 1 and lineage 3 where the epistatic effect of *pncB1* and *pncB2* with *pncA* was detected, especially with lineage 4 [23]. Whereas evidence suggests that the epistatic relationship with *pncA* is weaker than *pncB2*. In the current study samples, a majority have *pncA* mutations (Supplementary Files S1) also harboring mutations in *pncB1* or *pncB2*.

Although synonymous mutations are commonly considered to be without phenotypic effect, there is growing evidence that these mutations may affect gene expression and protein folding, ultimately providing an adoptive favor to the organism [24,25,26,27]. In very few cases, the mechanisms of synonymous mutations on organism phenotypic effect have been illuminated. Zwart et al. identified 10 synonymous mutations in TEM-1 β-lactamase which increased the *Escherichia coli* resistance to cefotaxime [24]. Moreover, synonymous mutations may have an important effect as the application of specific codons may increase the transgene expression by 1000-fold [28]. Codon usage may be different even within a single gene. Synonymous mutations at some particular locations may have some experience selection because they interrupt motifs of proteins that are recognized by post and pre-transcriptional regulatory mechanisms, which happens in microRNAs that require ribosomal pausing sites for proper folding or modification processes in ubiquitin [29].

Predicting the effect of mutations on thermodynamic stability (ΔΔG) might be important in protein science. To gain functional insight into the amino acid substitution and its effect on protein function, molecular dynamic simulation in combination with bioinformatics tools might be important for initial observations [30]. All the non-synonymous mutations in *pncB1* exhibited a stabilizing effect (Figure 1) which may be in favor of bacterial growth and survival in extreme conditions. However, this effect needs further validation through laboratory mutant experiments for a better understanding of the effect on MTB growth and survival.

## 5. Conclusions

All the drug-resistant samples harbored mutations in *pncB1* or *pncB2*. Mutations at position Pro447Ser, Arg286Arg, Gly127Ser, and delTCAGGCCG 1499213>1499220 were novel in *pncB1*. The non-synonymous mutations exhibited stabilizing effects on the *pncB1* structure. Mutations detected in *pncB2* were all synonymous except one, which may increase the gene expression level or protein folding effect. However, further studies are needed to elucidate the effect of these synonymous mutations on organism phenotype. The presence of mutations in *pncB1* and *pncB2* of all drug-resistant isolates may be linked with phenotypic drug susceptibility testing with a large number of genomic isolates for a better understanding of the phenomena of associations among mutations in genes. Inhibitors based on the most common *pncB1* and *pncB2* mutants may be useful in high TB burden countries.

## Figures and Tables

**Table 1:** Mutations in *pncB1* gene of *Mycobacterium tuberculosis*.

Position	Nucleotide Change	Type of Mutation		Amino Acid Change
1499213	T	Del	* GAP	
1499214	C	Del	GAP	
1499215	A	Del	GAP	
1499216	G	Del	GAP	
1499217	G	Del	GAP	
1499218	C	Del	GAP	
1499219	C	Del	GAP	
1499220	G	Del	GAP	
1499221	G	SNP	A	* Pro447Ser (ccg/Tcg)
1499274	C	SNP	G	Gly429Ala (ggc/gCc)
1500201	G	SNP	A	Ser120Leu (tca/tTa)
1499702	C	SNP	G	* Arg286Arg (cgg/cgC)
1500181	C	SNP	T	* Gly127Ser (ggc/Agc)

* Novel.

**Table 2:** Mutations detected in *pncB2* gene of *Mycobacterium tuberculosis* isolates.

Sample	Position	Wild Type	Mutant	Amino Acid Change
737	666631	A	G	Phe204Phe (ttt/ttC)
741	666631	A	G	Phe204Phe (ttt/ttC)
752	666742	C	T	Ala167Ala (gcg/gcA)
754	666631	A	G	Phe204Phe (ttt/ttC)
767	666631	A	G	Phe204Phe (ttt/ttC)
770	666631	A	G	Phe204Phe (ttt/ttC)
790	666631	A	G	Phe204Phe (ttt/ttC)
797	666742	C	T	Ala167Ala (gcg/gcA)
801	666631	A	G	Phe204Phe (ttt/ttC)
802	666631	A	G	Phe204Phe (ttt/ttC)
* ERR2510337	666275	G	A	^#^ Ala323Val (gcg/gTg)
* ERR2510358	666387	A	C	^#^ Phe286Val (ttc/Gtc)

* Supplementary file S2, ^#^ Novel.

## Data Availability

The genomic data in the current study are available in NCBI under accessions (ERX3360434-ERX3360514, ERR2510337-ERR2510445, ERR2510546-ERR2510645).

## Supplementary Materials

The following supporting information can be downloaded at: https://www.mdpi.com/article/10.3390/polym14081623/s1, Supplementary files S1: Mutational data of retrieved files (ERX3360434-ERX3360514, ERR2510337-ERR2510445, ERR2510546-ERR2510645); Supplementary file S2: Mutations in *pncB1* and *pncB2*.
